# High waist circumference rather than high body mass index may be a predictive risk factor for the longer disease duration of chronic spontaneous urticaria

**DOI:** 10.1038/s41598-021-81484-1

**Published:** 2021-01-21

**Authors:** Yeong Ho Kim, Kyung Do Han, Chul Hwan Bang, Ji Hyun Lee, Jun Young Lee, Yong Gyu Park, Young Min Park

**Affiliations:** 1 Department of Dermatology, Seoul St. Mary’s Hospital, College of Medicine, The Catholic University of Korea, 222 Banpo-daero, Seocho-gu, Seoul, 06591 Republic of Korea; 2 Department of Statistics and Actuarial Science, Soongsil University, Seoul, Republic of Korea; 3 Department of Biostatistics, College of Medicine, The Catholic University of Korea, Seoul, Republic of Korea

**Keywords:** Skin diseases, Risk factors

## Abstract

In a previous study, we found that higher waist circumference (WC) and higher body mass index (BMI) both increase the risk of chronic spontaneous urticaria (CSU). The aim of this study was to determine whether WC and BMI also increase the duration of CSU. We used multivariable Cox proportional hazards models to determine the hazard ratio (HR) for longer disease duration (longer than 3 years) according to WC and BMI. A total of 52,667 subjects were enrolled and their mean age was 54.5. After adjustments for other confounding variables the high WC/high BMI group had 1.062 times higher HR (95% CI, 1.028–1.098) than the normal WC/normal BMI group. Interestingly, the high WC/normal BMI group showed a significantly higher HR (1.053; 95% CI, 1.008–1.101) than the normal WC/normal BMI group, but not the normal WC/high BMI group (0.998; 95% CI, 0.951–1.046). Taken together, our results suggest that high WC rather than high BMI is a predictive risk factor for the longer disease duration of CSU.

In a previous study, we found that higher waist circumference (WC) and higher body mass index (BMI) both increase the risk of chronic spontaneous urticaria (CSU)^1^. Another population-based study from Italy found a positive correlation between CSU risk and obesity defined by BMI^2^. A population-based study of the natural course of CSU in Korea found that the 1-, 2-, 3-, 4-, and 5-year remission rates of CSU were 21.5%, 33.0%, 38.9%, 42.6%, and 44.6%, respectively^3^. A retrospective observational study of 549 CSU patients in Spain revealed that the percentage of patients who experienced disease durations of 1 year, 3 years, 5 years, and more than 5 years were 64.1%, 32.4%, 17.3% and 8.4%, respectively^4^. Several risk factors such as the presence of autoantibodies, age older than 45 years, and vitamin D deficiency have been reported to be associated with longer duration of CSU^4–7^. A study of 85 CSU patients in Poland revealed that higher BMI values had a trend towards longer disease duration^8^.

The aim of the current study was to determine whether WC and/or BMI also increase the duration of CSU. We conducted a nationwide, cross-sectional, population-based study using data from the Korean National Health Insurance Service (NHIS). The NHIS is a national health care program covering the entire Korean population. This study was approved by the Ethics Committee of Seoul St. Mary’s Hospital, the Catholic University of Korea (KC17ZESI0487), and was conducted according to the principles of the Declaration of Helsinki. The need of the written informed consent has waived by the ethics committee of Seoul St. Mary's Hospital, the Catholic University of Korea. The follow-up period was from January 2009 to December 2015. We used the following diagnosis-based algorithm to identify CSU patients: patients who had visited outpatient clinics twice at least 6 weeks apart and received 2 diagnoses designated by the International Classification of Disease (ICD)-10 code for idiopathic urticaria (L50.1), other specified urticaria (L50.8), or unspecified urticaria (L50.9), or patients who had visited outpatient clinics twice at least 6 weeks apart with one diagnosis of L50.1, L50.8, or L50.9 plus another diagnosis of angioneurotic edema (T78.3)^1,9^. Considering the total follow-up period of 7 years, we defined the incidence rate of longer disease duration as the percentage of patients with disease durations of longer than 3 years among the total CSU patients. We followed-up CSU patients identified by the diagnosis-based algorithm for at least 3 years after the first detection to determine whether they could be identified with the CSU diagnosis-based algorithm again, until December 2015. The clinical characteristics (sex, age, WC, BMI, smoking, alcohol, exercise, residential area, household income, diabetes mellitus, hypertension, and hyperlipidemia) of CSU patients were analysed using Student’s t-test and the chi-square test. Normal BMI was defined as < 25 kg/m^2^ and normal WC was defined as < 90 cm in male or < 85 cm in female, according to the Korean definition for obesity^10^. As WC and BMI are not always directly proportional to each other, we divided CSU patients into 4 groups as follows: normal WC/normal BMI, normal WC/high BMI (≥ 25 kg/m^2^), high WC (≥ 90 cm in male or ≥ 85 cm in female)/normal BMI, and high WC/high BMI. We used these categories to determine the impacts of WC and/or BMI on the duration of CSU. We used multivariable Cox proportional hazards models to determine the hazard ratio (HR) for longer duration CSU according to WC and BMI after adjustments for confounding variables. *P* < 0.05 was considered to be statistically significant. We performed statistical analyses using SAS version 9.4 (SAS Institute, Cary, NC, USA).

A total of 52,667 CSU patients were enrolled in this study. Their mean age was 54.5. Number of patients in 20–30, 40–64, 65–79, and ≥ 80 years age groups were 7883 (15.0%), 30,122 (57.2%), 11,686 (22.2%), and 2976 (5.7%), respectively. Number of female patients was 28,632 (54.4%). 22,215 patients (42.18%) were found to have longer disease duration (> 3 years). Female patients had a significantly higher incidence rate of longer duration (42.62%) than male patients (41.66%) (*P* < 0.05). Patients equal or above 80 years old had the highest incidence rate of longer disease duration (47.08%), followed by patients aged 65–79 (44.70%), 40–64 (42.45%) and 20–39 (35.57%). Patients with high WC (44.59%) and high BMI (43.43%) also had significantly higher incidence rates of longer disease duration, compared with normal WC patients and normal BMI patients, respectively (*P* < 0.05). Other factors that were associated with significantly higher incidence rates of longer disease duration were residential area (rural) (43.39%), diabetes mellitus (DM) (44.2%), hypertension (44.2%), and hyperlipidemia (44.2%) (*P* < 0.05), but not smoking, alcohol, exercise, or household income.

After adjustments for age, gender, comorbidities of diabetes mellitus, hypertension, and hyperlipidemia, there was no significantly higher HR in each BMI group, but two WC groups (90/85 ≤ WC < 95/90, 95/90 ≤ WC < 100/95) showed significantly higher HRs (1.056; 95%CI, 1.006–1.109, 1.082; 95% CI, 1.016–1.152, respectively) (Table 1). After adjustments for the same variables above, the high WC/high BMI group had 1.062 times higher HR (95% CI, 1.028–1.098) than the normal WC/normal BMI group. Interestingly, the high WC/normal BMI group showed a significantly higher HR (1.053; 95% CI, 1.008–1.101) than the normal WC/normal BMI group, but the normal WC/high BMI group did not (0.998; 95% CI, 0.951–1.046) (Table 2).

**Table 1:** Multivariable Cox proportional hazards models to determine the HR for longer duration CSU according to WC and BMI.

Group	Total no.	Event^a^	Incidence rate^b^	HR (95% CI)	
				Model 1	Model 2
BMI (kg/m^2^)					
BMI < 18.5	1702	691	203.215	1.018 (0.942–1.101)	1.019 (0.942–1.103)
18.5 ≤ BMI < 23	19,049	7792	202.202	1 (reference)	1 (reference)
23 ≤ BMI < 25	13,645	5797	212.211	1.026 (0.992–1.062)	1.012 (0.974–1.052)
25 ≤ BMI < 30	16,438	7122	217.688	1.048 (1.015–1.082)	1.007 (0.963–1.052)
BMI ≥ 30	1833	813	226.962	1.103 (1.026–1.186)	1.041 (0.951–1.14)
WC (male/female, cm)					
WC < 80/75	15,743	6334	195.7	1 (0.963–1.038)	1.005 (0.965–1.047)
80/75 ≤ WC < 85/80	12,025	4958	203.65	1 (reference)	1 (reference)
85/80 ≤ WC < 90/85	11,581	4985	216.969	1.037 (0.997–1.079)	1.032 (0.991–1.076)
90/85 ≤ WC < 95/90	7592	3351	225.706	1.065 (1.019–1.113)	1.056 (1.006–1.109)
95/90 ≤ WC < 100/95	3676	1669	236.012	1.097 (1.037–1.160)	1.082 (1.016–1.152)
WC ≥ 100/95	2050	918	232.636	1.073 (1.000–1.152)	1.042 (0.957–1.134)

*BMI* body mass index, *CI* confidence interval, *CSU* chronic spontaneous urticarial, *HR* hazard ratio, *WC* waist circumference.

^a^Event; disease duration over 3 years, ^b^per 1000 person-years.

Data were expressed as HR and 95% CI.

Model 1 was adjusted for age and gender.

Model 2 was adjusted for age, gender, comorbidities of diabetes mellitus, hypertension, and hyperlipidemia.

**Table 2:** Subgroup analysis of on the longer duration of CSU.

WC	BMI	Total no.	Event^a^	Incidence rate^b^	HR (95% CI)	
					Model 1	Model 2
Normal	Normal	28,448	11,616	201.094	1 (reference)	
Normal	High	5111	2091	198.520	1.002 (0.955–1.050)	0.998 (0.951–1.046)
High	Normal	5936	2660	231.997	1.061 (1.015–1.108)	1.053 (1.008–1.101)
High	High	13,150	5837	226.718	1.072 (1.038–1.107)	1.062 (1.028–1.098)

*BMI* body mass index, *CI* confidence interval, *CSU* chronic spontaneous urticarial, *HR* hazard ratio, *WC* waist circumference.

High WC was defined as ≥ 90 cm in males or ≥ 85 cm in females, and high BMI was defined as ≥ 25 kg/m^2^.

^a^Event; disease duration over 3 years, ^b^per 1000 person-years.

Data were expressed as HR and 95% CI.

Model 1 was adjusted for age and gender.

Model 2 was adjusted for age, gender, comorbidities of diabetes mellitus, hypertension, and hyperlipidemia.

Zbiciak-Nylec et al^8^ firstly reported that higher BMI values had a trend towards longer disease duration. In this study, we further revealed that higher WC rather than higher BMI is more related with the longer disease duration of CSU, although both WC and BMI can affect the disease duration. Nevertheless, the pathophysiology of causal relationship between obesity and CSU has not been fully elucidated so far. According to previous studies, adipokines may act on human mast cells to induce endothelial inflammation^11^. As endothelial inflammation progresses, atherosclerotic changes proceed^12^. In the atherosclerotic plaque mast cells can be activated by variable proinflammatory factors such as C3a, C5a, cytokines, monocyte chemoattractant protein-1 and oxidized low-density lipoprotein^13^. Activation of mast cells through this process may affect the development of CSU^8^. Our results that especially, high WC/normal BMI group showed a higher risk for a longer disease duration of CSU but the normal WC/high BMI group did not, may reflect that those with high WC/normal BMI may have relatively high fat mass while those with normal WC/high BMI may have relatively high muscle mass.Taken together, these results suggest that high WC rather than high BMI is a predictive risk factor for the longer disease duration of CSU.
